# Hypoglycemia and Death in Mice Following Experimental Exposure to an Extract of *Trogia venenata* Mushrooms

**DOI:** 10.1371/journal.pone.0038712

**Published:** 2012-06-20

**Authors:** GuoQing Shi, Jun He, Tao Shen, Robert E. Fontaine, Lin Yang, ZhongYu Zhou, Hong Gao, YanFeng Xu, Chuan Qin, ZhuLiang Yang, JiKai Liu, WenLi Huang, Guang Zeng

**Affiliations:** 1 Chinese Field Epidemiology Training Program, Chinese Center for Disease Control and Prevention, Beijing, China; 2 The Institute of Laboratory Animal Science, Chinese Academy of Medical Sciences and Peking Union Medical College, Beijing, China; 3 United States Centers for Disease Control and Prevention, Atlanta, Georgia, United States of America; 4 Yunnan Institute of Endemic Diseases Control and Prevention, Dali, Yunnan Province, China; 5 State Key Laboratory of Phytochemistry and Plant Resources in West China, Kunming Institute of Botany, Chinese Academy of Sciences, Kunming, Yunnan Province, China; The University of Texas at San Antonio, United States of America

## Abstract

**Background:**

Clusters of sudden unexplained death (SUD) in Yunnan Province, China, have been linked to eating *Trogia venenata* mushrooms. We evaluated the toxic effect of this mushroom on mice.

**Methods:**

We prepared extracts of fresh *T. venenata* and *Laccaria vinaceoavellanea* mushrooms collected from the environs of a village that had SUD. We randomly allocated mice into treatment groups and administered mushroom extracts at doses ranging from 500 to 3500 mg/kg and water (control) via a gavage needle. We observed mice for mortality for 7 days after a 3500 mg/kg dose and for 24 hours after doses from 500 to 3000 mg/kg. We determined biochemical markers from serum two hours after a 2000 mg/kg dose.

**Results:**

Ten mice fed *T. venenata* extract (3500 mg/kg) died by five hours whereas all control mice (*L. vinaceoavellanea* extract and water) survived the seven-day observation period. All mice died by five hours after exposure to single doses of *T. venenata* extract ranging from 1500 to 3000 mg/kg, while the four mice exposed to a 500 mg/kg dose all survived. Mice fed 2000 mg/kg of *T. venenata* extract developed profound hypoglycemia (median  = 0.66 mmol/L) two hours after exposure.

**Discussion:**

Hypoglycemia and death within hours of exposure, a pattern unique among mushroom toxicity, characterize *T. venenata* poisoning.

## Introduction

Since the late 1970’s, over 300 apparently healthy persons in north western central Yunnan Province died suddenly of unexplained causes [1]. These sudden unexplained deaths (SUD) occurred in remote villages in a 60,000 km^2^, 22 county areas [1]. Ninety percent of SUD clusters occurred in July and August [2], [3], [4]. SUD affected young to middle aged adults more common with mean age of 33 (1–83 years) [2], and women had a 1.6-fold higher risk than men [4]. In 2005 case-control studies identified picking mushrooms as a risk factor. However, the symptoms including palpitation, dizziness, chest distress, shortness of breath, abdominal pain, syncope or temporary loss of consciousness preceding the deaths and the suddenness of the deaths were unlike those expected from any known mushroom toxin [1], [2], [3], [4]. Moreover, no specific mushroom was identified.

During 2006–2009, we identified five SUD clusters in which the victims had recently eaten mushrooms of the genus *Trogia*, which was not known to contain toxic species [1]. These mushrooms were distinct from described species of *Trogia* and have subsequently been described as a new species, *Trogia venenata* (Mycobank registration number MB 561711) [5]. Following the epidemiologic findings, we carried out toxicity studies of extracts of *T. venenata* harvested from the environs of an affected village.

## Materials and Methods

### Ethics Statement

No specific permits were required for the described field studies. The locations that we collected mushrooms are not privately-owned or protected in any way. Permission is not required for collecting wild mushrooms to eat, sell or for research in open and public mountain areas in China. This field collection did not involve any endangered or protected species.

### Specimen Preparation

We collected 500 g fresh material of each of *T. venenata* and *Laccaria vinaceoavellanea*, a common edible mushroom from the forest around a village in Tengchong county, Yunnan Province. This village had two SUD in 2006 and SUD clusters had occurred in other nearby villages in other years. A mushroom taxonomist from Kunming Institute of Botany, Chinese Academy of Sciences, identified *T. venenata* and *L. vinaceoavellanea.*

We transported the mushrooms from the field to the laboratory within 15 hours at ambient temperature (about 26°C). Upon arrival at the laboratory we soaked each mushroom type in 1000 ml 70% ethanol for 24 hours at 40°C three times. We then combined the three ethanol extracts and evaporated the ethanol yielding 13.5931 g of *T. venenata* extract and 12.9436 g *L. vinaceoavellanea* extract. Each extract was in a gel phase. The ratio of extract to fresh mushrooms was 1∶36.8 for *T. venenata* and 1∶38.6 for *Laccaria* mushrooms.

### Experimental Mice

We used as experimental animals clean Kunming mice (originated from SPF mice) supplied from Beijing HFK Bio-technology Co. Ltd. Mice were 5–7 weeks old and weighed about 20 g each. For each sex, the range around the mean weight was ±20%. The mice were quarantined for 2–3 days after delivery to the laboratory.

All experimental procedures on animals were according to the guidelines of the Association for the Assessment and Accreditation of Laboratory Animal Care International (AAALAC) in our AAALAC-accredited animal facility. The Institutional Animal Care and Use Committees (IACUC), Institute of Laboratory Animal Science, Chinese Academy of Medical Sciences & Peking Union Medical College specifically approved this study (approval ID N-09-2002).

Before exposure, the mice were fasted for 16 hours. They were then weighed and assigned to experimental exposure groups described below. All exposures were administered as an aqueous solution containing 100 mg of extract per mL into the stomach through a gavage needle (4–5 cm long and 1 mm diameter with metal ball at the tip). All exposure doses of extract were based on the weight of the individual mice and expressed as mg (of extract)/kg.

### Acute Toxicity Testing

After stratification by sex, the mice were assigned at random into two groups of 10 mice each (five male and five females). We administered doses of 3500 mg/kg of *T. venenata* extract to mice in one group and of 3500 mg/kg *L. vinaceoavellanea* extract mice in the other. Mice that survived five hours after the first dose received a second 3500 mg/kg dose of the same extract. We observed the mice for general activity and signs of possible toxicity until death or for survivors for seven days.

### Fatal Dose Range

We assigned the mice at random after stratification by sex to five groups by dose of *T. venenata* extract. We gave 3000 mg/kg body weight of extract to eight mice and 2500 mg, 2000 mg, 1500 mg and 500 mg/kg body weight of extract to groups of four mice each. We used the fixed-dose method to determine the LD 50 of the *T. venenata* extract. We estimated the exposure doses to an average 70 kg human as follows:

1. Total intake (mg) of fresh mushroom in mice  =  Total intake (mg) of extract [Exposure dose (mg/kg body weight/time) × body weight (kg) of mice × number of times of administration] × ratio of extract to fresh mushrooms;
2. Estimated grams of fresh mushroom for a human  =  Total intake (mg) of fresh mushroom in mice × body surface area equivalent coefficient of mice and human (387.9 for 20 g mice and 70 kg weight of human) ÷ 1000.

### Serum Biochemistry, Enzymes, and Electrolytes

After stratification by sex, we assigned mice at random to three groups of 20 mice (ten female and ten male). One group was given the 2000 mg/kg of *T. venenata* extract. A second group, *L. vinaceoavellanea* controls, received 2000 mg/kg of *L. vinaceoavellanea* extract. The third, water control, received an equivalent volume of distilled water. Two hours after exposure we anesthetized the mice with a sodium pentobarbital injection, collected blood from the retro-orbital sinus, and sacrificed the mice with additional sodium pentobarbital.

We separated the serum from the blood. We used a 7020 automatic biochemical analyzer to determine: alanine aminotransferase, aspartate aminotransferase, total protein, albumin, globulin, albumin to globulin ratio, alkaline phosphatase; urea nitrogen, creatinine, uric acid, glucose, high density lipoprotein cholesterol, low density lipoprotein cholesterol, triglycerides, total cholesterol, lactate dehydrogenase, potassium, sodium, calcium and chlorine.

### Data Analysis

The Fisher’s exact test was used to assess the significance of differences among experimental groups for categorical data of acute toxicity and t-test for mean value comparison of serum biochemistry, enzymes, and electrolytes. We used Log Rank test for survival data and Spearman’s rank sum test to assess the relationship between groups for biochemical data. All tests of significance were two-sided and p<0.05 was considered statistically significant.

## Results

### Acute Toxicity

30 minutes (range: 20–115 min) after exposure to *T. venenata* (Mycobank registration number MB 561711) [5] extract (3500 mg/Kg. body weight), all ten mice developed signs of toxicity. Initially, spontaneous activity decreased (100%), followed by no activity (70%), tremor (40%), intermittent seizures (20%), tachypnea (10%) or hypopnea (10%).

Between two to three hours after feeding extracts, seven (70%) mice died. After a second exposure, five hours after the initial exposure, the three surviving mice died (6 h, 7.6 h and 23 h after the initial exposure). The ten *L. vinaceoavellanea*-control mice received two feedings of 3500 mg/kg of *L. vinaceoavellanea* extract 5 h apart. All ten survived the full seven-day post-exposure observation period. None developed any signs of illness or toxicity during that period. The differences between the *T. venenata*-exposed and the control group for both illness and mortality were statistically significant (*P*<0.001, Fisher’s exact test).

### Fatal Dose Range

All mice in the groups exposed to 1500 to 3000 mg/kg of *T. venenata* extract developed signs of illness whereas the group exposed to the lowest dose (500 mg/kg) did not. All affected mice had reduced spontaneous activity, followed by inactivity 12 (75%), difficulty breathing six (38%), convulsions three (19%), tremors, tachypnea two (13%), and intermittent jerking one (6%). The median time from feeding to onset of first sign of toxicity was 24 min (range: 5 min–41 min). Death occurred spontaneously in 16 of the 16 mice 2.2 to 4.4 h after exposure in the four higher dose (1500 to 3000 mg/kg) groups (Table 1). The survival time was shorter (median 2.4 h) in the 3000 mg/kg group (p<0.001; Log Rank test) than in the other three groups. All four mice that received the lowest dose (500 mg/kg) survived the seven-day observation period. The doses in terms of fresh *T. venenata* mushrooms were from 370 mg to 2200 mg; approximately equivalent to a human eating from 140 g to 860 g. The minimum fatal dose fell between 370 mg–1100 mg of fresh mushrooms; approximately equivalent to a human eating 140 g–430 g (Table 1).

**Table 1:** Mortality of mice exposed to an extract of *Trogia venenata* mushrooms by dose of extract.

	Fresh mushroom equivalent in grams		Mice		Hours from exposure to death		
Extract Dose(mg/Kg)	Mice*	Human†	Exposed	Deaths	Median	Min.	Max.
3000	2.21	856	4	4	2.4	2.2	3.0
2500	1.84	713	4	4	3.7	3.2	4.4
2000	1.47	571	4	4	3.3	2.6	3.8
1500	1.10	428	4	4	3.3	3.2	4.4
500	0.37	143	4	0	–	–	–

* Total intake (mg) of fresh mushroom in mice  =  Total intake (mg) of extract [dose of extract (mg/kg body weight/time) × body weight (kg) of mice × times of administration] × Ratio of extract to fresh mushroom (36.8).

† Estimated total fresh mushroom intake in human (g)  =  Total intake (mg) of fresh mushroom in mice × body surface area equivalent coefficient of mice and human (387.9 for 20 g mice and 70 Kg weight of human) ÷ 1000.

### Serum Enzymes, Metabolites, and Electrolytes

The greatest difference between *T. venenata*-exposed mice, *L. vinaceoavellanea* controls, water controls, or reference values for laboratory mice was a profound hypoglycemia (mean  = 1.63 mmol/L; median  = 0.66 mmol/L) in the *T. venenata*-exposed mice (Table 2). The higher mean than median value of glucose in *T. venenata*-exposed mice resulted from two outliers (6.7 and 4.6 mmol/L) whereas the remaining 10 serum glucose values ranged from 0.09 to 2.2 mmol/L. Elevation of serum triglycerides, low density lipoproteins cholesterol also appeared in *T. venenata*-exposed mice but not in control mice. We observed an inverse relationship of triglycerides to glucose (r = −0.91, p<0.01; Spearman’s rank sum test). Weaker inverse trends for low density lipoproteins cholesterol to glucose (r = −0.51) were not statistically significant. Among the other serum chemistries *T. venenata*-exposed mice had lower blood urea nitrogen and a higher total protein than either control group. All other determinations of serum biochemistries, enzymes, and electrolytes did not show statistically significant differences between *T. venenata*-exposed mice and *Laccaria*-controls, water-controls, or reference values. In particular, we observed no indication of liver or kidney dysfunction.

**Table 2:** Mean serum biochemical determinations* two hours after feeding mice with extracts (2000 mg/Kg B.W.) of *Trogia venenata*, *Laccaria vinaceoavellanea*, or water (control).

	Exposure	T. venenata	Water control	L. vinaceoavellanea	Difference**	*p* ††
GLU† (mmol/L)	N	12	20	20		
	Mean	1.63	7.41	7.21	4.5×↓	<0.0001
	Sd	2.03	2.55	2.62		
	Median	0.66	7.63	7.59		
	Range	0.09–6.70	2.36–11.93	2.15–11.41		
TG‡ (mmol/L)	N	8	20	20		
	Mean	2.14	0.8	0.99	2.7×↑	<0.0001
	Sd	1.16	0.21	0.46		
	Median	2.01	0.84	0.85		
	Range	0.71–4.26	0.52–1.25	0.60–2.48		
LDL-C§(mmol/L)	N	15	20	19		
	Mean	0.16	0.09	0.07	1.8×↑	<0.001
	Sd	0.08	0.04	0.07		
	Median	0.15	0.1	0.08		
	Range	0.05–0.35	0–0.15	0–0.19		
TP‖ (g/L)	N	13	20	20		
	Mean	59.04	54.48	57	1.08×↑	<0.05
	Sd	6.33	4.46	3.95		
	Median	58.62	55.21	56.2		
	Range	49.01–69.66	47.65–62.09	51.13–66.25		
BUN¶(mmol/L)	N	13	20	20		
	Mean	5.72	9.07	9.94	1.6×↓	<0.01
	Sd	1.49	3.3	2.93		
	Median	5.82	9.5	10.22		
	Range	3.07–8.81	3.19–14.30	5.13–15.20		

* Only determinations with statistically significant differences between *T. venenata* and water controls are shown. Differences determinations of serum electrolytes, metabolites, and enzymes between *T. venenata* exposed and water control were not statistically significant for all other biochemical, enzyme, and electrolyte tests including cholesterol, high density lipoproteins, uric acid, creatinine, globulin, albumin, albumin to globulin ratio, alkaline phosphatase, alanine aminotransferase, aspartine aminotransferase, lactic dehydrogenase, potassium, sodium, calcium, and chloride.

† GLU, glucose.

‡ TG, triglycerides.

§ LDL-C, low density lipoproteins.

‖ TP, total protein.

¶ BUN, blood urea nitrogen.

** Relative difference (increase↑, decrease↓) between *T. venenata*-exposed and water-exposed mice.

†† t-test comparing mean values of *T. venenata*-exposed and water-exposed mice.

## Discussion

This experiment demonstrated acute, fatal toxicity in mice from an extract of *T. venenata*. The toxic compounds in *T. venenata* extract have since been isolated and identified as 2 amino acids (2R-amino-4S-hydroxy-5-hexynoic acid and 2R-amino-5-hexynoic acid) that differ by one hydroxyl group [6]. Hypoglycemia in humans and experimental animals results in coma if serum glucose is under 2.7 mmol/L and brain death if below 1.0 mmol/L [7]. In humans, sudden death from hypoglycemia is also attributed to cardiac arrhythmias [7]–[10]. We found no other biochemical abnormality that could explain the death of the mice. Accordingly, the profound hypoglycemia (median  = 0.66 mmol/L) observed following exposure to *T. venenata* extract was alone sufficient to explain the death of the mice in our experiment.

Hypoglycemia below 1.0 mmol/L is extremely rare in humans, and it usually results from insulin overdose or insulin producing tumors. However, an edible plant, the ackee fruit, produces acute hypoglycemia with measured serum glucose below 1.0 mmol/L and associated fatalities [11]. Ackee fruit contains two toxic amino acids, hypoglycin A and hypoglycin B, that block beta oxidation of fatty acids and gluconeogenesis [11]. Without alternatives to beta oxidation and glucose as an energy source, rapid depletion of circulating glucose and glycogen stores results. Additionally, carnitines, fatty acids, and other lipids accumulate in the liver, blood, and urine. The pattern of toxicity from *T. venenata* also had one other similarity to hypoglycins, an increase in serum fatty acids in proportion to the hypoglycemia. Both toxic amino acids of *T. venenata* and hypoglycin have a structural similarity, a δ-unsaturated bond [6]. Follow-on toxicological studies will be needed to define the precise toxic mechanism of *T. venenata*, confirm the similarities, and identify differences with hypoglycin poisoning.

Other mushroom toxins that can lead to hypoglycemia are amatoxins and gyromitrin and are found in other genera than *Trogia* [12]. Hypoglycemia associated with both amatoxins and gyromitrin is secondary to hepatic necrosis, 2–7 days after exposure, and follows gastrointestinal, renal and hepatic manifestations of toxicity. The *T. venenata*-associated hypoglycemia, in contrast, occurred within 2 hours of exposure in the absence of elevated serum transaminases or renal dysfunction. The degree of hypoglycemia (0.66 mmol/L) that we observed from *T. venenata* extract was far greater than expected for either of these two toxins. Fatal doses of amatoxins are below 1 mg/kg, equivalent to a human eating a single mushroom; a far lower toxic dose than we observed for *T. venenata*.

The minimum fatal dose range in these mice was equivalent to a human eating 140–430 g of fresh *T. venenata* mushrooms. Another estimate using the toxic amino acids from *T. venenata* gives a similar amount [6]. They are also consistent with histories of victims of five clusters SUD who ate estimated totals of 400 g of *T. venenata* 5–9 times over a few days and an estimated 1500 g in 7 days [1]. The toxicity was also characterized by a moderate to high dose of extract and a relatively narrow threshold between a non-toxic and highly fatal dose.

This study has several limitations. We did not anticipate a toxin that deprived cells of their main energy substrate, glucose. Accordingly, we did not measure additional indicators of abnormal glucose, carbohydrate, or lipid metabolism. A characteristic of Yunnan SUD was the high rate of underlying heart lesions that we hypothesized increased susceptibility to a toxin. We could not replicate these field conditions since we used only normal mice. Because mice could not be fed sufficient amounts of mushroom to replicate typical amounts eaten by humans, we relied on an extract to expose mice and to assure uniform dosages among exposure groups. Alcohol extraction is an accepted methodology for mushroom toxicity studies, and has not been shown to modify toxic substances from other mushrooms. Nonetheless, unexpected changes in the toxin could result from mushroom preparation in these villages or from the extraction. The finding of one of the toxic amino acids in the postmortem heart blood of one victim suggests that alteration of the toxin was probably not occurring [6].

Although the full characterization of the toxicity of this mushroom is incomplete, these toxicity studies add strong support to our current health education campaign in Yunnan. Warnings to villagers about eating unfamiliar mushrooms in 2006 and *T. venenata* in 2008 were followed by a 73% decline in SUD through 2009 with no clusters of SUD in 2010 and 2011 in intervention villages [1]. Advising treatment of future poisoning with *T. venenata* with parenteral glucose needs to be done with precautions. If done early it will probably be effective and not harmful. However, experimental and clinical evidence suggests that glucose given to patients with hypoglycemic coma may provoke neuronal death [13].
